# *Allium macrostemon* whole extract ameliorates obesity-induced inflammation and endoplasmic reticulum stress in adipose tissue of high-fat diet-fed C57BL/6N mice

**DOI:** 10.29219/fnr.v67.9256

**Published:** 2023-05-18

**Authors:** Juhae Kim, Joo-Yeon Lee, Choon Young Kim

**Affiliations:** 1 Research Institute of Human Ecology, Yeungnam University, Gyeongsan, Gyeongbuk 38541, South Korea; 2 Department of Food and Nutrition, Yeungnam University, Gyeongsan, Gyeongbuk 38541, South Korea.

**Keywords:** obesity, Allium macrostemon extract, adipose tissue, inflammation, endoplasmic reticulum stress

## Abstract

**Background:**

Obesity is a major risk factor for metabolic syndrome and a serious health concern worldwide. Various strategies exist to treat and prevent obesity, including dietary approaches using bioactive ingredients from natural sources.

**Objective:**

This study aimed to investigate the anti-obesity effect of whole-plant *Allium macrostemon* (also called as long-stamen chive) extract (AME) as a potential new functional food.

**Design:**

C57BL/6N mice were divided into three groups and fed either a control diet (CD), high-fat diet (HFD), or HFD with AME treatment (200 mg/kg BW daily) for 9 weeks. The mice in the CD and HFD groups were treated with vehicle control.

**Results:**

AME supplementation reduced HFD-induced body weight gain, fat mass, and adipocyte size. AME suppressed peroxisome proliferator-activated receptor γ and fatty acid synthase mRNA expression, indicating reduced adipogenesis and lipogenesis in adipose tissue. In addition, AME lowered inflammation in adipose tissue, as demonstrated by the lower number of crown-like structures, mRNA, and/or protein expression of macrophage filtration markers, as well as pro-inflammatory cytokines, including F4/80 and IL-6. Endoplasmic reticulum stress was also alleviated by AME administration in adipose tissue. Several phenolic acids known to have anti-obesity effects, including ellagic acid, protocatechuic acid, and catechin, have been identified in AME.

**Conclusion:**

By suppressing adipose tissue expansion and inflammation, AME is a potential functional food for the prevention and/or treatment of obesity and its complications.

## Popular scientific summary

- This is the first report demonstrating that *Allium Macrostemon* extract (AME) from its entire plant exerts anti-obesity effect in high-fat diet-induced obese mice.
- AME supplementation attenuated adipose tissue expansion and inflammation in high-fat diet-induced obese mice.
- The beneficial anti-obesity and anti-inflammatory activities of AME were possibly mediated through regulating adipogenesis and inhibiting endoplasmic reticulum stress.

Obesity is a major public concern due to the increased risk of chronic non-communicable diseases, including dyslipidemia, cardiovascular diseases, and type 2 diabetes (1). The main pathological feature of obesity is excessive fat accumulation in adipose tissue, which involves an increase in the size (hypertrophy) and the number of adipocytes (hyperplasia) (2). The expansion of adipocytes results from the alteration of lipid metabolism in adipose tissue, including adipogenesis and lipogenesis. Adipogenesis, the differentiation of adipocyte precursor cells into adipocytes, requires CCAAT/enhancer-binding protein (C/EBP) family members and peroxisome proliferator-activated receptor γ (PPARγ) (3). Lipogenesis involves fatty acid and triglyceride synthesis, and their enzymes include stearoyl-CoA desaturase 1 (SCD1) and fatty acid synthase (FASN) (4). Under conditions of excess fat accumulation, expanded adipocytes become dysfunctional and release various types of pro-inflammatory adipokines such as tumor necrosis factor-α (TNF-α), interleukin (IL)-6, and IL-1β (2). Adipocyte expansion also promotes an elevated frequency of adipocyte death and macrophage recruitment to adipose tissue. This adipocyte remodeling produces numerous ‘crown-like structures’ (CLS) consisting of macrophages surrounding dead adipocytes (5). A high prevalence of CLS is correlated with adipose tissue inflammation.

Endoplasmic reticulum (ER) stress has been proposed as one of the mechanisms underlying increased inflammation in obese adipocytes (6, 7). The ER is a cellular component in eukaryotic cells, where newly synthesized proteins are correctly folded and assembled (8). High metabolic demands, such as chronic nutrient overload, can impair ER homeostasis, resulting in ER stress. Under ER stress conditions, unfolded protein response signaling pathways are activated (9); therefore, their components are considered indicators of ER stress. These include increased expression of the spliced form of X-box-binding protein-1 (XBP1), activating transcription factor 4 (ATF4), C/EBP-homologous protein (CHOP), and upregulated phosphorylated levels of eukaryotic initiation factor 2 alpha (elF2α) and c-Jun N-terminal kinase (JNK) (7). A recent study revealed that a high-fat diet (HFD) intake resulted in the upregulation of CHOP in adipocytes and that CHOP-deficient mice showed improved HFD-induced macrophage infiltration. These results suggest that CHOP is a representative mediator linking ER stress and inflammation in the adipose tissue (10). JNK is also a mediator of ER stress and inflammation in adipose tissue since ER stress-induced JNK activation in obese adipocytes produces signaling pathways of insulin resistance and inflammation (11).

Numerous studies have indicated that edible and medicinal plants exhibit anti-obesity activities, resulting in little to no side effects (12, 13). *Allium macrostemon*, also known as long-stamen chive, has been widely grown for consumption and medical use in East Asia, including China, Japan, and Korea, but its health-promoting actions have yet to be scientifically studied (14). For medical purposes, only the bulbs of *Allium macrostemon* are used in a herbal medicine called Hae-Baek to treat cardiovascular disease in China (15). Whole-plant and leaf extracts of *Allium macrostemon* showed anti-adipogenic activity in 3T3-L1 preadipocytes, but bulb extracts of *Allium macrostemon* did not (16). Higher polyphenol contents were identified in whole-plant and leaf extracts of *Allium macrostemon* than in bulb extracts of *Allium macrostemon*. Although macrostemonoside A, a pure compound isolated from the bulb of *Allium macrostemon*, has been tested *in vivo* to demonstrate its remediation of the adverse effects of a HFD (17), the whole-plant extract of *Allium macrostemon* has not been studied in obesity research.

Plant extracts with anti-adipogenic activity commonly exhibit anti-obesity activity (18). In addition, whole-plant extracts may show superiority on beneficial biological activities compared to pure isolated compounds because of the synergistic effect of multiple polyphenols (19, 20). Furthermore, *Allium macrostemon* is generally consumed in its entirety. Therefore, the whole-plant *Allium macrostemon* extract (AME) containing both bulbs and leaves may have an anti-obesity effect. In this study, the effect of whole-plant AME on adipose tissue dysfunction, body weight, and composition were tested in mice fed a HFD. As ER stress may be an underlying mechanism of adipose tissue dysfunction, the ameliorating effect of AME on ER stress in adipose tissue was also examined.

## Materials and methods

### Preparation of AME

*Allium macrostemon* was purchased in April 2021 from a commercial market (Sacheon, Gyengnam, Korea). The dried part of the *Allium macrostemon* was extracted following previously reported procedures (21). Powdered *Allium macrostemon* was extracted with 10 volumes of water (g/L) using an autoclave (MaXterile 60, Daihan Scientific, Seoul, Korea) at 121°C for 20 min. After cooling, the solution was filtered through sterile gauze and centrifuged at 12,000 g for 20 min. The supernatant was then re-filtered through Whatman No. 1 filter paper, evaporated using a rotary evaporation concentrator (N-1300, Eyela, Tokyo, Japan), and freeze-dried. The yield was 54.8% of the initial dry weight of the entire *Allium macrostemon* plant. The obtained AME was stored at −20°C until use.

### Identification and quantification of phenolic compounds in AME

The phenolic compound profile of AME was determined using an Agilent 1200 high-performance liquid chromatography (HPLC) system (Agilent, Santa Clara, CA, USA) coupled with a diode array detector (DAD). Seven phenolic compounds (protocatechuic acid, catechin, *p*-coumaric acid, ellagic acid, chlorogenic acid, caffeic acid, and ferulic acid) and AME samples were separated using an Agilent Zorbax Eclipse XDB C18 column (4.6 × 250 mm × 5 μm, Agilent) at 27°C. The binary mobile phase consisted of water containing 0.1% formic acid (eluent A) and acetonitrile (eluent B). Elution was performed at a flow rate of 0.8 mL/min with the following gradient outline: 0–3 min, 5–12% solution B; 3–8 min, 12–14% solution B; 8–25 min, 14–20% solution B; 25–35 min, 20–35% solution B; 35–45 min, 35–60% solution B; 45–60 min, 60–100% solution B; 60–65 min, 100–5% solution B. The injection volume was 10 μL. UV/Vis spectra were recorded in the 190–700 nm range, and chromatograms were acquired at 280 and 320 nm. The components were identified based on the retention times, and UV spectra of the phenolic standards and their quantities were calculated using a calibration curve. The results were averaged and reported as micrograms per gram of dried AME.

### Experimental animals and diets

Nineteen male C57BL/6N mice (3-week-old), which are prone to diet-induced obesity, were purchased from Central Lab Animal (SLC, Osaka, Japan) and housed in ventilated cages under air-conditioned room at a temperature of 21 ± 2°C and a humidity of 50 ± 5% under a 12:12 light: dark cycle. After acclimatization for 2 weeks, the mice were randomly divided into three groups and fed the following diets for 9 weeks: control diet (CD, *n* = 6), high-fat diet (HFD, *n* = 6), or HFD + AME (*n* = 7). CD (containing 10 kcal% fat, 20 kcal% protein, and 70 kcal% carbohydrates; D12450B) and HFD (containing 60 kcal% fat, 20 kcal% protein, and 20 kcal% carbohydrates; D12452) were purchased from Research Diets, Inc. (New Brunswick, NJ, USA). The fat sources in the HFD were lard and soybean oil. Nutrient composition is shown in Table S1. AME dissolved in distilled water containing 0.01% dimethyl sulfoxide (Sigma-Aldrich, St. Louis, MO, USA) was administered at 200 mg/kg/day by oral gavage for 9 weeks (10 μL/g BW) to the HFD + AME group. An equal volume of the vehicle solution was simultaneously administered to mice in the CD and HFD groups. The mice were provided with food and water *ad libitum* throughout the study period. Body weight and food intake were measured twice and thrice per week, respectively. Energy intake was also calculated based on the food intake, wherein the energy consumed per gram were 3.85 kcal/g for CD and 5.24 kcal/g for HFD groups. The energy efficiency ratio (EER) was calculated as body weight gain per the amount of energy intake. The animal study protocol was approved by the Institute Institutional Animal Care and Use Committee of Yeungnam University (Approval NO. 2021-033).

### Oral glucose tolerance test

After 5 weeks of experimental diet feeding, six mice per group were 6-h-fasted and given a single dose of oral glucose (2 g/kg BW). Tail blood samples were analyzed for glucose concentration at 0, 15, 30, 60, 90, and 120 min after glucose loading using a glucometer (Accu-Chek®, Roche Diagnostic, Mannheim, Germany). The estimation of the rise in blood glucose during the test was calculated by applying the trapezoidal rule using the area under the curve.

### Measurement of body composition

One week before sacrifice, body composition analysis was performed using dual-energy X-ray absorptiometry (DEXA; iNSiGHT VET DXA; OsteoSys, Seoul, Korea). Three representative mice per group were selected and anesthetized with isoflurane (Isotroy 250; Troikaa Pharmaceuticals, Gujarat, India) during the scanning. In the color composition images, the fat and lean tissue areas are indicated as red and green, respectively.

### Serum analysis and tissue isolation

After 9 weeks of experimental period, all the mice were subjected to fasting for 16 h and anesthetized with isoflurane. Before tissue harvest, blood was collected by cardiac puncture into serum separation tubes (BD Microtainer ® tube, 365967, BD Biosciences, Franklin Lakes, NJ, USA), clotted for 30 min at RT, and centrifuged at 21,206 × g at 4°C for 90 s to isolate the serum. The serum was stored at −70°C until further analysis. Assay kits for determining serum levels of glucose (AM201-K), triacylglycerol (TG; AM-157S-K), total cholesterol (TC; AM-157S-K), and high-density lipoprotein cholesterol (HDL-C; AM-202-K) were purchased from Asan Pharmaceutical Co. (Seoul, Korea). Serum levels were determined using each commercial kit according to the manufacturer’s instructions. Low-density lipoprotein cholesterol (LDL-C) levels were calculated using the Friedewald equation (22). After collecting the blood, epididymal adipose tissue was rapidly removed from the mice and weighed immediately. The tissues were snap-frozen in liquid nitrogen and stored at −70°C until analysis.

### Histological analysis

Epididymal adipose tissue was fixed in a 10% neutral formalin solution and embedded in paraffin. Tissues were cut to a thickness of 6 μm and stained with hematoxylin and eosin (H&E). By using light microscopy (Eclipse Ni-U, Nikon, Tokyo, Japan) and an image analysis program (NIS-Element BR, Basic Research software, Nikon), the stained sections were analyzed to quantify the number and diameter of adipocytes and the number of CLS.

### RNA extraction, cDNA synthesis, and real-time polymerase chain reaction

Adipose tissue was homogenized using the TissueLyser system (85300, Qiagen, Venlo, Netherlands), and total RNA was isolated using the TRIzol reagent (15596018, Invitrogen Life Technologies, Carlsbad, CA, USA), according to the manufacturer’s instructions. The RNA content and purity were measured using a NanoDrop spectrophotometer (Thermo Fisher Scientific, Waltham, MA, USA). One microgram of total RNA was reverse-transcribed into cDNA using an AMPIGENE® cDNA synthesis kit (END-KIT106, Enzo Life Sciences, Farmingdale, NY, USA) and a SimpliAmp Thermal Cycler instrument (Applied Biosystems, Waltham, MA, USA). Real-time PCR analysis was performed on an AMPIGENE ® qPCR Green Mix Hi-ROX (ENZ-NUC104, Enzo Life Sciences) using a StepOne Plus real-time PCR system (Applied Biosystems). Mouse 18s rRNA was used as a reference gene, and the relative gene expression levels were analyzed using the 2^-∆∆^Ct method. The primers used for real-time PCR analysis are listed in Table S2.

### Protein extraction and western blotting analysis

The adipose tissue was homogenized in an ice-cold lysis buffer (100 mM Tris-HCl, pH 7.6, 100 mM NaCl, and 0.5% Triton X-100) containing 1 mM of sodium orthovanadate, 10 mM of sodium fluoride, and a protease inhibitor cocktail (P3100, GenDEPOT, Katy, TX, USA) using the TissueLyser system (Qiagen). Based on a previously published protocol (23), the homogenates were incubated at 4°C for 1 h with gentle rotation and centrifuged three times at 20,000 × g for 15 min at 4°C to remove excess lipids. The final supernatant was collected and stored at −70°C until further analysis. After quantification of the protein content of the lysates using the Pierce BCA protein assay kit (23225, Thermo Fisher Scientific), equal amounts of protein were loaded onto the lanes of an SDS-PAGE gel, separated, and transferred to a PVDF membrane (Millipore, Bedford, MA, USA). After being blocked with 5% non-fat milk (BD Biosciences) or bovine serum albumin (Bovostar, Bovogen, Victoria, Australia) in a Tris-buffered saline solution containing 0.05% Tween-20 (pH 7.5), the membrane was probed with a specific primary antibody (Table S3). The membrane was then incubated with a horseradish peroxidase-linked secondary antibody (Jackson ImmunoResearch, West Grove, PA, USA). The protein expression signal was detected using an HRP substrate (Dongin Bio, Seoul, Korea), and the immunoreactive bands were visualized and analyzed using the Amersham ImageQuant 800 (Cytiva, Marlborough, MA, USA). The expression of Heat shock cognate protein 70 (HSC70) was used as a control to monitor equal protein loading in each lane.

### Statistical analysis

The data was analyzed by one-way analysis of variance (ANOVA) followed by Tukey’s multiple comparison tests or by independent *t*-test using SPSS 27 Statistics (IBM, Chicago, IL, USA). All data are expressed as mean ± standard error of the mean (SEM), except for the quantification of phenolic compounds in Table 1, where the data are presented as mean ± standard deviation. *P* was set at less than 0.05. Some data points with *P* values between 0.05 and 0.10 were considered to have ‘trend’ to be differed between or among groups evaluated. The interquartile range (IQR) of the serum analysis dataset was used to identify outliers. The IQR was calculated as the third quartile (Q3) minus the first quartile (Q1). Values falling below Q1–1.5 IQR or above Q3 + 1.5 IQR were treated as outliers and excluded from the analyses.

**Table 1:** Quantitative analysis of phenolic compounds in *Allium macrostemon* whole extract

Compound	Retention time (min)	Content^1^ (μg/g of extract)
Protocatechuic acid	8.90^2^	268.49 ± 2.96
Catechin	11.33^2^	216.66 ±12.42
*p*-Coumaric acid	30.65^2^	42.00 ± 0.06
Ellagic acid	42.80^2^	295.90 ± 0.69
Chlorogenic acid	10.80^3^	113.23 ± 0.17
Caffeic acid	14.35^3^	17.60 ± 0.32
Ferulic acid	24.08^3^	197.88 ± 0.36

1 Data is shown as the mean ± SD (*n* = 2).

2 The detector wavelength was 280 nm.

3 The detector wavelength was 320 nm.

## Results

### Characterization of AME

The AME was analyzed by HPLC-DAD to identify and quantify polyphenolic compounds present in *Allium* species (24). A total of seven compounds were identified in the AME: four were detected at 280 nm, and three were detected at 320 nm (Table 1 and Fig. S1). Based on the regression equations derived from the chromatogram of each standard material, the amounts of each phenolic compound identified in the AME were in the range of 17.6 to 295.9 μg/g of AME and ranged from most to least abundant as follows: ellagic acid > protocatechuic acid > catechin > ferulic acid > chlorogenic acid > *p*-coumaric acid > caffeic acid.

### AME reduced body weight and fat mass in HFD-induced obese mice

**Fig. 1:**
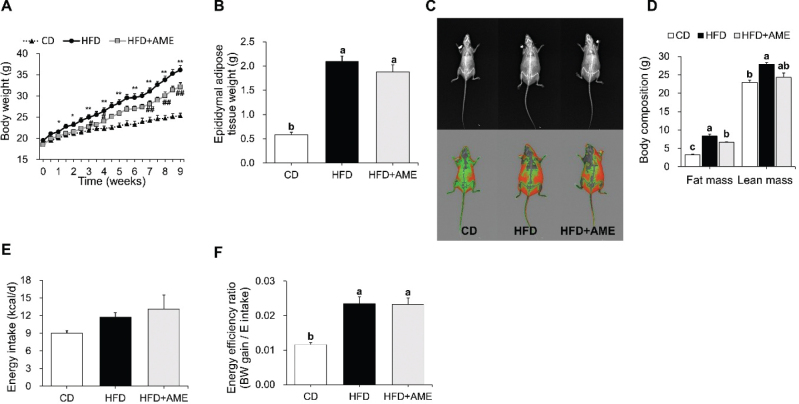
Effects of *Allium macrostemon* extract supplementation on HFD-induced body weight, adipose tissue weight, body composition, and energy intake. (A) Body weight changes during the experimental period. *P < 0.05 and **P < 0.001 between CD and HFD groups, ^#^*P* < 0.05 and ^##^*P* < 0.001 between HFD and HFD + AME groups. (B) Epididymal adipose tissue weight. (C) Representative images of mice obtained by DEXA. The upper images are X-ray images, and the lower images are color composition images indicating fat (red) and lean (green) mass. (D) Calculated fat and lean mass obtained by DEXA. (E) Energy intake. (F) Energy efficiency ratio. Data are expressed as the mean ± SEM and assessed by one-way ANOVA followed by Tukey’s multiple comparison test (*P* < 0.05). Different letters (a, b and c) indicate significant difference among treatment. ‘a’ stands for the highest value, while ‘c’ stands for the lowest. DEXA, dual-energy X-ray absorptiometry; CD, control diet; HFD, high-fat diet; HFD + AME, high-fat diet supplemented with *Allium macrostemon* extract; ANOVA, analysis of variance; SEM, standard error of the mean.

After 9 weeks of experimental diet feeding, the body and epididymal adipose tissue weight of the HFD group increased by 1.47 and 2.60 times, respectively, compared to the CD group. The AME-supplemented group showed a significant decrease in body weight compared to the HFD group (ANOVA, *P* < 0.001 for body weight and *P* < 0.001 for epididymal adipose tissue weight) (Fig. 1A). Although there is no significant difference in epididymal depot mass between HFD and HFD + AME groups (Fig. 1B), AME-induced reduction in whole-body adiposity was observed by whole-body composition based on DEXA scanning (Fig. 1C). Quantification of whole-body fat mass also demonstrated that the HFD-induced increased in fat mass (159%) was significantly reduced by 20% in the HFD + AME group (ANOVA, *P* < 0.001) (Fig. 1D). Energy intake was also evaluated, but no significant differences were observed among the groups (Fig. 1E). Consistent with body weight gain, the EER was higher in the HFD group than that in the CD group. No additional differences between the AME-supplemented and the HFD and CD groups were observed (Fig. 1F).

### AME tended to decrease glucose intolerance in HFD-induced obese mice

**Fig. 2:**
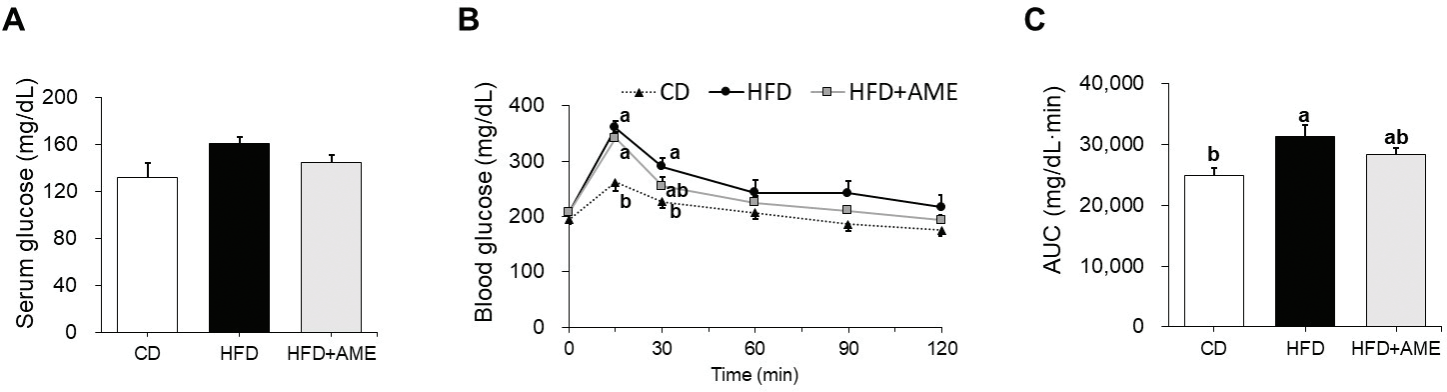
Effects of AME supplementation on serum glucose level and glucose tolerance in HFD-fed mice. (A) Serum glucose level at sacrifice. (B) Changes in blood glucose levels during the oral glucose tolerance test. (C) AUC of the glucose tolerance test. Data is expressed as the mean ± SEM and assessed by one-way ANOVA followed by Tukey’s multiple comparison test (*P* < 0.05). Different letters (a and b) indicate significant difference among treatment. ‘a’ stands for the highest value, while ‘b’ stands for the lowest. AUC, area under the curve; CD, control diet; HFD, high-fat diet; HFD + AME, high-fat diet supplemented with *Allium macrostemon* extract; ANOVA, analysis of variance; SEM, standard error of the mean.

Since a saponin isolated from the bulb of *Allium macrostemon* lowered hyperlipidemia and hyperglycemia in HFD-fed mice (17), the effect of the whole extract of *Allium macrostemon* on serum lipid and glucose levels was investigated. No significant differences in serum levels of TG, TC, HDL-C, LDL-C, or the HDL-C to TC ratio (HTR) were noted among the groups (Table S4). Serum glucose levels were higher in the HFD group than in the CD group (Indepedent-*t*-test, CD vs. HFD, *P* = 0.070), and lower in the HFD+AME group than in the HFD group (Indepedent-*t*-test, HFD vs. HFD + AME, *P* = 0.072), but ANOVA analysis showed no significant differences among the groups (Fig. 2A). Likewise, AME-supplemented mice displayed better glucose disposal ability than non-AME-supplemented HFD-fed mice (Figs. 2B and C).

### AME ameliorated HFD-induced adipocyte expansion

**Fig. 3:**
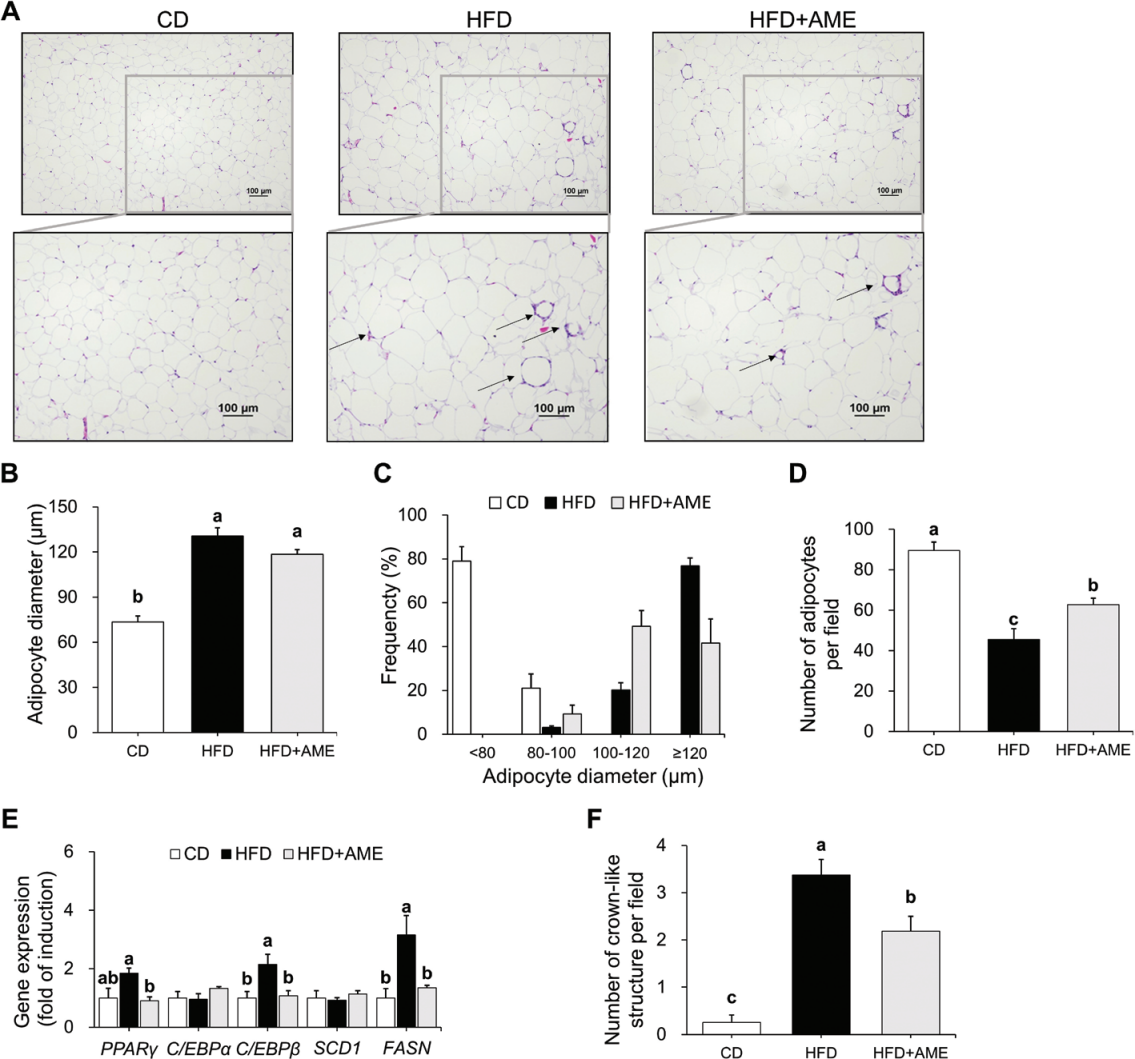
Effects of AME supplementation on HFD-induced adipose tissue morphological changes and related mRNA gene expressions. (A) Representative hematoxylin and eosin staining of epididymal adipose tissue (100 × magnification, scale bar = 100 μm). CLS are depicted with arrows in the lower figures. (B) Mean adipocyte diameter. (C) Distribution of adipocyte diameter. (D) Number of adipocytes per field. (E) Relative mRNA levels of adipogenesis (PPARγ, C/EBPα and C/EBPβ) and lipogenesis (SCD1 and FASN) marker genes. (F) Number of CLS counted under one field in 100× magnification. Data is expressed as the mean ± SEM (*n* = 3 for histological analysis, *n* = 4 for PCR analysis) and assessed by one-way ANOVA followed by Tukey’s multiple comparison test (*P* < 0.05). Different letters (a, b and c) indicate significant difference among treatment. ‘a’ stands for the highest value, while ‘c’ stands for the lowest. CLS, crown-like structures; CD, control diet; HFD, high-fat diet; HFD + AME, high-fat diet supplemented with *Allium macrostemon* extract; ANOVA, analysis of variance; SEM, standard error of the mean.

To determine whether the body weight-reducing effect of AME was due to a decrease in fat accumulation, morphological changes in adipose tissue were evaluated by H&E staining (Fig. 3A). The adipocyte size of the HFD group (80% of adipocyte diameter was below 80 μm) was larger than that of CD group (80% of adipocyte diameter exceeded 120 μm). AME supplementation decreased adipocyte size, with smaller and fewer large adipocytes compared to the HFD group (ANOVA, *P* < 0.001) (Figs. 3B and C). Similarly, an increased number of cells per field was observed in the HFD + AME group compared to that in the HFD group (ANOVA, *P* < 0.001) (Fig. 3D). The mRNA expression of adipogenic genes, including *PPARγ* (1.85-fold induction) and *C/EBPβ* (4.45-fold induction), as well as the lipogenic gene *FASN* (3.15-fold induction), was consistently upregulated by HFD; whereas, AME supplementation inhibited gene expression upregulated by HFD, resulting in 0.49-fold (*PPARγ*), 0.28-fold (*C/EBPβ*), 0.43-fold (*FASN*) reduction, respectively (Fig. 3E). In addition to alterations in adipocyte size, distinctive levels of inflammation in adipocytes were detected by observing CLS in each group. The HFD group showed a significantly higher number of CLS than the CD group, whereas the HFD + AME group showed a 35% lower number of CLS than the HFD group (ANOVA, *P* < 0.001) (Fig. 3F).

### AME attenuated adipose tissue inflammation in HFD-induced obese mice

**Fig. 4:**
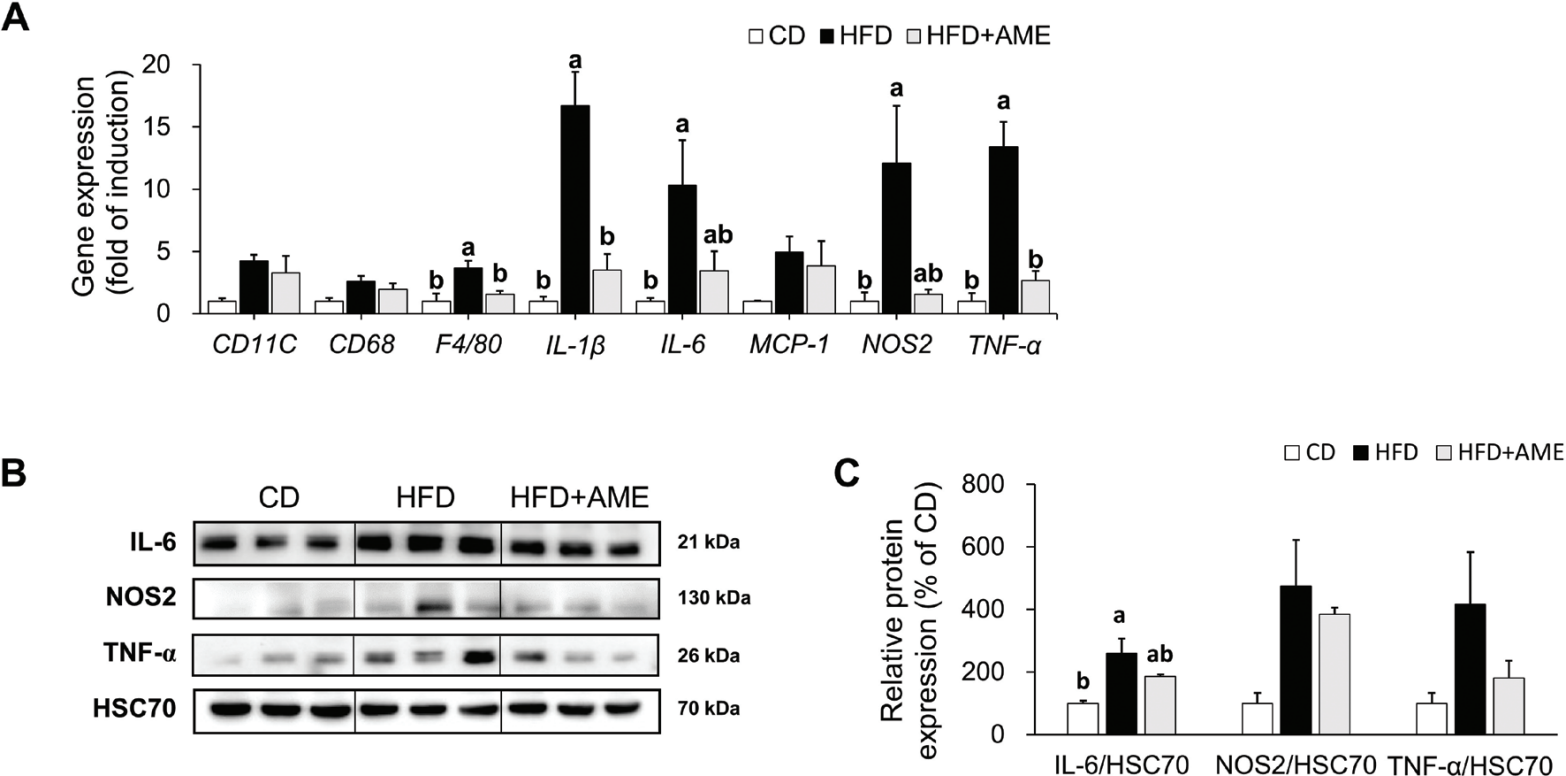
Effects of AME supplementation on HFD-induced adipose tissue inflammation. (A) Relative mRNA levels of inflammation markers genes. (B, C) Relative protein levels of IL-6, NOS2, and TNF-α. Data is expressed as the mean ± SEM (*n* = 4 for PCR analysis, *n* = 3 for western blotting analysis) and assessed by one-way ANOVA followed by Tukey’s multiple comparison test (*P* < 0.05). Different letters (a and b) indicate significant difference among treatment. ‘a’ stands for the highest value, while ‘b’ stands for the lowest. CD, control diet; HFD, high-fat diet; HFD + AME, high-fat diet supplemented with *Allium macrostemon* extract; ANOVA, analysis of variance; SEM, standard error of the mean.

The anti-inflammatory effects of AME supplementation on the adipose tissue of HFD-fed mice, as shown in Fig. 3F, were also confirmed by mRNA and protein analyses. The mRNA levels of macrophage markers, such as F4/80 (0.42-fold reduction), as well as pro-inflammatory cytokines, including IL-1β (0.21-fold reduction), IL-6 (0.33-fold reduction), nitric oxidase synthase 2 (NOS2, 0.13-fold reduction), and TNF-α (0.20-fold reduction) tended to be lower in the HFD + AME group than in the HFD group (ANOVA, *P* < 0.001 for IL-1β and *P* < 0.001 for TNF-α) (Fig. 4A). The protein levels of IL-6, NOS2, and TNF-α were also lower in the HFD + AME group than those in the HFD group, resulting in 0.72-fold, 0.81-fold, and 0.43-fold reduction, respectively (Figs. 4B and C).

### AME reduced adipose tissue ER stress in HFD-induced obese mice

**Fig. 5:**
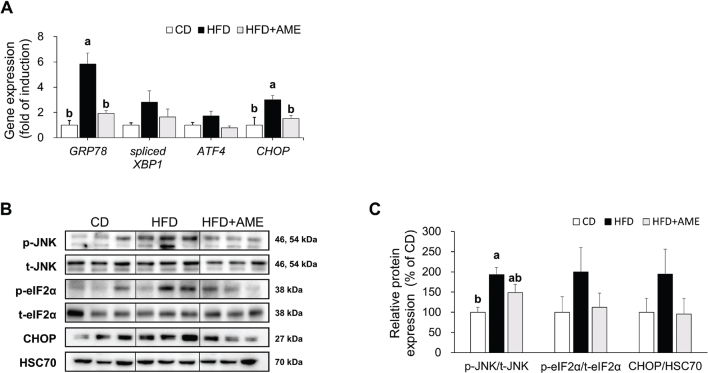
Effects of AME supplementation on HFD-induced adipose tissue ER stress. (A) Relative mRNA levels of ER stress marker genes. (B, C) Relative protein levels of phosphorylated JNK, phosphorylated eIF2α, and CHOP. Data is expressed as the mean ± SEM (*n* = 4 for PCR analysis, *n* = 3 for western blotting analysis) and assessed by one-way ANOVA followed by Tukey’s multiple comparison test (*P* < 0.05). Different letters (a and b) indicate significant difference among treatment. ‘a’ stands for the highest value, while ‘b’ stands for the lowest. CD, control diet; HFD, high-fat diet; HFD + AME, high-fat diet supplemented with *Allium macrostemon* extract; ANOVA, analysis of variance; SEM, standard error of the mean.

The expression levels of the genes and proteins involved in ER stress activation were examined. The mRNA expression of the ER stress chaperone glucose-regulated protein 78 (GRP78) and ER stress effector CHOP in the HFD group were significantly higher than those in the CD group by 5.8-fold and 3.0-fold, respectively. These increases were significantly inhibited by 66.9 and 49.2% in the HFD + AME group (ANOVA, *P* < 0.001 for GRP78) (Fig. 5A). Phosphorylation of JNK, a marker for activation of the inositol-requiring enzyme 1 (IRE1), branch of ER, and inflammatory signaling, was upregulated in the HFD group by 1.9-fold compared to the CD group. The HFD + AME group showed a 0.77-fold lower phosphorylation level than the HFD group (Figs. 5B and C). Despite no statistical difference, the phosphorylated eIF2α and CHOP levels in the HFD group showed increases of 2.0-fold and 1.9-fold, respectively, compared to the CD group. The expression in the HFD + AME group tended to be suppressed by 44 and 50%, respectively, compared to the HFD group.

## Discussion

Adipocyte expansion and inflammation are the two major features of adipose tissue in obesity. Substantial evidence has shown that food extracts can attenuate adipose tissue dysfunction, suggesting their potential as therapeutic interventions for the prevention and treatment of obesity (25–27). Whole-plant AME has been shown to suppress the adipogenesis of preadipocytes by decreasing the expression of adipogenic and lipogenic genes in an *in vitro* 3T3-L1 cell model (16). However, the effect of whole-plant AME on diet-induced obesity *in vivo* remains unknown. Therefore, this study examined the anti-obesity effects of AME in HFD-fed mice. The results demonstrate that AME reduced HFD-induced body weight gain and adiposity. Furthermore, the anti-obesity activity of AME is accompanied by decreased adipocyte hypertrophy and the reduced expression of genes and proteins related to adipogenesis, lipogenesis, inflammation, and ER stress.

Accumulating evidence suggests that the inhibition of adipogenesis and lipogenesis moderates the anti-obesity effects of natural plant-derived food components (12, 13). Previous work demonstrated that AME treatment in 3T3-L1 cells inhibits mRNA expression of adipogenesis regulators, including *C/EBP*β, *PPAR*γ, and *C/EBPα*, compared to untreated controls (16). The downregulation of *PPARγ* and *C/EBPβ* mRNA expression levels in the HFD + AME group compared to those in the HFD group was observed, which was consistent with the *in vitro* data. As a key regulator of adipocyte differentiation, downregulation of PPAR*γ* may imply a reduced capacity for adipogenesis, leading to a reduction in adipose tissue mass (28). Supplementation with *Allium fistulosum L.* (green or welsh onion) extract at a dose of 400 mg/kg/day for 6.5 weeks also resulted in reduced body weight gain and smaller adipocyte size in HFD-induced obese mice, with suppression of the mRNA expression of *PPARγ* in adipose tissue (29). Concerning lipogenesis, AME supplementation decreased the mRNA level of *FASN*, which synthesizes fatty acid from acetyl-CoA and malonyl-CoA *de novo* and is recognized as a potential functional food targeting obesity-related health risks (30). Diallyl trisulfide, which is a thioester and a major flavor compound in *Allium* vegetables, has been reported to lower the activity of FASN in 3T3-L1 cells (31). Ellagic acid, which was reported to reduce the mRNA levels of *PPARγ* and *FASN* in differentiated 3T3-L1 adipocytes (32), was identified as the highest phenolic compound in AME during the HPLC analysis of this study. The increased gene expression of *FASN* in adipose tissue is associated with enlarged epididymal fat mass and an increase in insulin resistance and pro-inflammatory cytokines (33). These associations could elucidate the current findings that AME supplementation reduced fat mass and inflammation as shown in Figs. 1D and 4.

Adipose tissue-derived production of pro-inflammatory factors underlies the link between obesity and its associated metabolic complications (6). As presented in Fig. 3D and 4, this study showed that AME supplementation had inhibitory effects on HFD-induced inflammation, as detected by protein and gene expression levels of pro-inflammatory markers, including IL-6 and NOS2, as well as histological examinations of the CLS structures. Previously, Wu et al. (34) investigated the anti-inflammatory effect of AME in the rat model of vascular endothelial injury. Compared to the control, AME-treated rats had decreased mRNA expression levels of the inflammatory genes, such as cyclooxygenases (COX-1 and -2) and nitric oxide synthases (iNOS/NOS2 and eNOS) (34). The *Allium sativum* organosulfur compounds, alliin and 1,2-vinyldithiin, decreased the secretion and expression of inflammatory cytokines such as IL-6 and monocyte chemoattractant protein-1 in human preadipocytes and 3T3-L1 adipocytes (35, 36). In recent studies, using both *in vitro* and *in vivo* models, AME-derived organosulfur compounds, such as diallyl trisulfide and diallyl disulfide, manifested apparent antioxidant activity (37), which may be associated with an anti-inflammatory function (38).

AME supplementation was demonstrated to inhibit ER stress, which is a mediator of adipose tissue inflammation (9, 11). ER stress induces the dissociation of the ER chaperone GRP78 from the three ER sensor branches, PERK, IRE1, and ATF6, which sequentially activate the downstream signaling cascade (8). The JNK signaling pathway, a downstream target of the IRE1 branch, is activated by phosphorylation and induces the production of several pro-inflammatory cytokines in adipocytes, thereby contributing to pro-inflammatory macrophage infiltration (39). Moreover, the three branches of the unfolded protein response result in CHOP transcription (40) which is linked to inflammation. CHOP-deficient mice showed improved HFD-induced macrophage infiltration (10). AME supplementation alleviated ER stress in HFD-fed mice, as reflected by the reduced expression of *GRP78* and *CHOP* mRNA and phosphorylated JNK protein. Similar to AME, other plant extracts, especially *Allium* species, also exhibit anti-inflammatory action under obese subject conditions. For example, *Allium cepa L*. (white onion) (41) and *Allium satvium* (black garlic) (42) extracts showed anti-inflammatory properties in a diet-induced obese model. In addition, administration of *Panax notoginseng* (Chinese ginseng) extract for 4 weeks exerted anti-obesity activity by suppressing ER stress and inflammation in the adipose tissue of HFD-fed mice. To the best of our knowledge, this study is the first to show the inhibitory effects of AME administration on inflammation and ER stress in a diet-induced obese rodent model. The role of ER stress in adipogenesis and lipogenesis in obesity has been well-explained in a recent review (43). Over-activation in adipose tissue ER stress can aggravate lipogenesis by all three ER sensor branches, mainly via sterol regulatory element-binding protein 1c (SREBP1c). Adipogenesis is regulated by the IRE1 pathway via C/EBPα. AME has a clear inhibitory effect on ER stress by downregulating the gene and protein expression levels related to ER stress. In addition, the altered lipogenic expression of SREBP1c as *FASN* gene was detected downstream as well as the adipogenic expression of *PPARγ* and *C/EBPβ*. Considering the role of ER stress as a molecular process in the pathogenesis of obesity-associated metabolic disorders, the inhibition of ER stress is one of the major mechanisms for the anti-obesity effects of AME.

Even though epididymal adipose tissue weight was not significantly different between the HFD and HFD + AME groups, other fat pads may be different, as suggested by the DEXA data. The fat masses detected using DEXA are composed of several depots, such as epididymal, mesenteric, peritoneal, and cardiac depots. In addition to these visceral adipose tissues, subcutaneous interscapular and inguinal depots were calculated for the fat masses (44). Since no change in mass was observed in the epididymal fat depot between the HFD and HFD + AME groups, the AME treatment decreased the weights of other depots, such as perirenal or subcutaneous depots rather than epididymal depots. Likewise, different responses to the HFD on each fat depot have been reported. In one study, weights of the perineal fat depot, but not the epididymal fat depot, were increased after 20 weeks of the HFD in C57BL/6 mice (45). In another study, while subcutaneous and mesenteric fat depot mass was increased, the epididymal fat depot mass was reduced after 12 weeks of the HFD in C57BL/6 mice (46). Although the tendency of reduction in lean mass composition by absolute value was observed in HFD + AME group (HFD: 27.9 ± 0.4 g vs. HFD + AME: 24.3 ± 1.2 g), relative lean mass to body weight is comparable between the two groups (HFD: 75.3 ± 1.0% vs. HFD + AME: 74.6 ± 2.8%). Thus, the possibility to inhibit normal healthy growth by AME treatment can be ruled out. Moreover, there was a decrease in lean mass relative to body weight in the HFD group compared to the CD group (CD: 85.9 ± 1.0% vs. HFD: 75.3 ± 1.0%). Similarly, previous studies have reported that HFD feeding reduced lean mass (%) in a mouse model using short-term (6 weeks) (47) or long-term (21 weeks) (48) protocols.

Serum levels of TG and TC typically increase due to HFD. However, contradictory results have also been reported. A study using HFD with 60% kcal from fat showed that 2 weeks of feeding did not induce changes in TG and TC levels, yet a further 6 weeks of feeding decreased TG levels with no changes in TC. HFD also increases adiposity (49). These results suggest that changes in serum lipid levels may take longer than changes in tissue size and that increased serum lipid levels may not necessarily be accompanied by diet-induced obesity. Another HFD feeding study which was conducted for up to 12 weeks, suggested that the reduced TG level might result from HFD-induced partitioning of lipids into the liver (50). No significant alterations in serum TG and TC levels were reported in an 8-week HFD feeding study (60% kcal from fat) (51) or a 12-week HFD feeding study (60% kcal from fat) (52) that corresponded to the findings in this study. Moreover, mice might not be an appropriate model to study serum lipid metabolism. A previous study investigated comprehensive plasma lipid profile to identify appropriate preclinical models using 24 different species including mice and revealed that non-human primate and dog are compatible models with dyslipidemic human (53). The other study also demonstrated the strongest similarities in lipid fingerprints between human and hamster and in lipoprotein profiles between human and pig (54). The major difference between human and mice is lipoprotein profiles. While mice display markedly lower LDL-C levels than HDL-C levels, human profile is characterized by elevated LDL-C levels than HDL-C levels (53, 54).

Previously the total polyphenol content was reported as 14 mg gallic acid equivalent per gram (GAE/g) of AME (16). This amount was higher than that in *Allium sativum L.* stem extract (6 mg GAE/g of extract), which ameliorated HFD-induced obesity and insulin resistance in mice supplmented with 250 mg extract/kg diet for 4 weeks (55). In this study, the individual polyphenol content of AME is examined further. Some individual phenolic compounds identified in AME have been reported to exhibit anti-obesity activity. Ellagic acid and protocatechuic acid, approximately 295.9 and 268.5 μg/g of AME, respectively, are described in Table 1. Ellagic acid, the predominant phenolic compound in AME, is a naturally occurring polyphenol with anti-obesity activity. Pure ellagic acid or plant extracts rich in ellagic acid attenuated adipocyte expansion in both *in vitro* and *in vivo* models (56). In addition, protocatechuic acid, the second most abundant phenolic compound in AME, is one of the main metabolites of anthocyanins with antioxidant and anti-inflammatory activities. These biological functions are related to the attenuation of obesity and related metabolic disorders (57). Lastly, the other phenolic compounds detected in AME, including catechin (58), ferulic acid (59), chlorogenic acid (60), *p*-coumaric acid (61) and caffeic acid (62), have been demonstrated to have anti-obesity effects in HFD-fed mouse studies. When combined, these findings present promising individual bioactive compounds, such as ellagic acid and protocatechuic acid, which might explain the anti-obesity effect of AME in the HFD-fed mice model.

There are two studies that attempted to absorption-distribution-metabolism-excretion (ADME) of AME, one *in vivo* and the other *in silico* method. In rat model, a Chinese herbal medicine named Wen-Yang-Huo-Xue formula containing AME at 21% of total composition was orally administrated and rat plasma was analyzed for pharmacokinetics of active compounds including ginsenoside Rb1, ginsenoside Rg1, paeoniflorin, albiflorin and oxypaeoniflorin. The last three saponins were absorbed rapidly with peak time within 1 h and were eliminated quickly (63). In the other study, seven active constituents of AME including six types of steroidal saponins such as smilagenin, gitogenin, and macrostemonoside D as well as one flavonoid, naringenin, were evaluated for the ADME properties. The *in silico* analysis revealed the seven compounds displayed high oral bioavailability (35–53.5%), long half-life (≥ 4h), and high Caco-2 permeability, which reflect the delivery to the systemic circulation, the timescale of eliciting therapeutic effect, and the rate and extent of absorption, respectively (64). Although these two studies examined with AME, polyphenols were not investigated. ADME properties of polyphenols from *Allium cepa L*. waste have been recently reported (65, 66). The high theoretical absorption was observed for protocatechuic acid (89.15%) and ellagic acid (60.2%). The protocatechuic acid also showed the highest Caco-2 permeability and was only polyphenol considered to cross the blood-brain barrier in aspect of distribution. In terms of metabolism, ellagic acid was considered as a CYP1A2 inhibitor, meaning the potential reduction in the biotransformation of drugs. Taken together, studies on pharmacokinetics of AME-derived bioactive phenolic compounds after taken up with HFD like our model setting have not been tested. Nevertheless, as discussed above, the ellagic acid and protocatechuic acid, which are abundant in AME based on our HPLC analysis (Table1), may elicit high bioavailability in mice. Further studies are needed to determine these bioactive phenolic compounds in serum or tissue to obtain supporting evidence.

This is the first study to report the inhibitory effect of whole-plant AME on HFD-induced body weight gain. In addition, a possible mechanism for the anti-obesity effect was explored and subsequently changes in inflammation and ER stress in the adipose tissue were found. Conducting a clinical trial to test the anti-obesity activity of AME would be the next step toward developing functional food materials from AME at a clinical level. However, there are some limitations in this study. One of the limitations of this study was that only male mice were used with a relatively short experimental period of 9 weeks. Since female rodents are relatively resistant to HFD-induced responses such as hyperphagia and weight gain and are protected from short-term HFD-induced alterations in energy balance (67), male mice were used in the present study. In future studies, testing females and a longer feeding period must be considered. In addition, only one dose of 200 mg/kg BW was tested in this study. Testing different concentrations may generate more distinct effects on glucose intolerance, which did not show significant effectiveness in the present study. Last but not least, there were no changes in serum lipid parameters in the present HFD-feeding study using mice model. Since the mice has little human compatibility in plasma lipid and lipoprotein metabolism (53, 54), the uncertainty regarding the potential for AME on lowering lipid levels might need to be elucidated in the future study using other preclinical models or clinical model.

## Conclusion

This study provides scientific evidence to support the anti-obesity function of AME in HFD-fed mice by focusing on adipose tissue dysfunction. The results of this study demonstrated that whole-plant AME supplementation, daily at 200 mg/kg BW, significantly reduced HFD-induced body weight gain, especially adipose tissue hypertrophy. In addition, AME ameliorated HFD-induced obese adipose tissue disorders such as elevated inflammation and ER stress, indicating the ability of AME to attenuate obesity-related metabolic disorders. Thus, AME is a potential candidate ingredient in functional foods to reduce and manage obesity and obesity-related diseases.
