# Supplementary material for: Allium macrostemon whole extract ameliorates obesity-induced inflammation and endoplasmic reticulum stress in adipose tissue of high-fat diet-fed C57BL/6N mice
Source: Food Nutr Res. 2023 May 18;67:10.29219/fnr.v67.9256. doi: 10.29219/fnr.v67.9256 (PMC10202093; doi:10.29219/fnr.v67.9256)
Supplement: Supplementary file 1 [file FNR-67-9256-s001.docx]

***Fig. 1.***

***
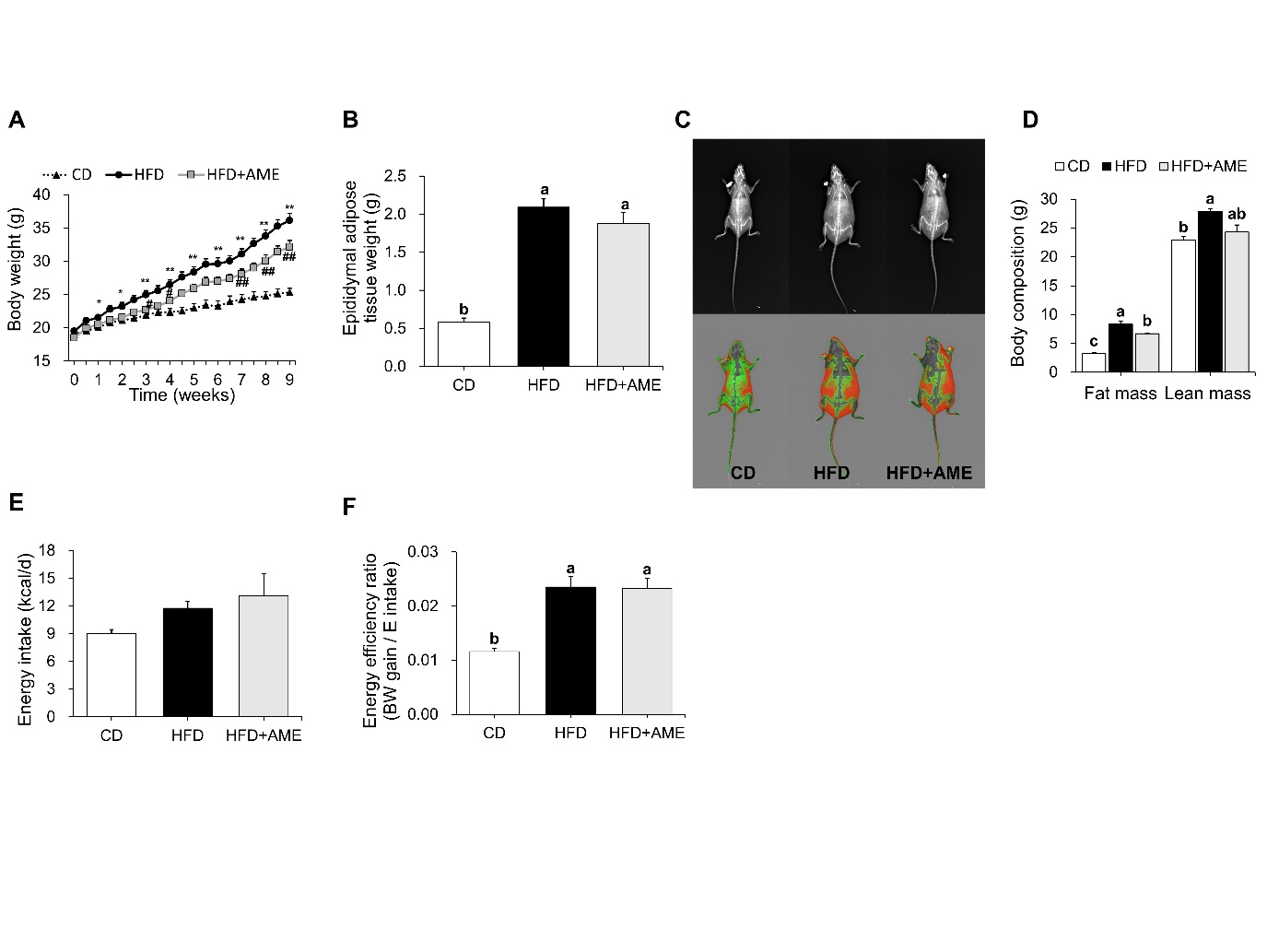
***

**Table S2.** **Primer sequences used in quantitative real-time PCR**

| **Gene**^1^ | | **Forward (5′→3′)** | **Reverse (5′→3′)** |
| --- | --- | --- | --- |
| *Adipogenesis* | *CEBPα* | GGT TTA GGG ATG TTT GGG TTT TT | AAG CCC ACT TCA TTT CAT TGG T |
|  | *CEBPβ* | AGC GGC TGC AGA AGA AGG T | GGC AGC TGC TTG AAC AAG TTC |
|  | *PPARγ* | CCC ACC AAC TTC GGA ATC AG | AAT GCT GGA GAA ATC AAC TGT GGT A |
| *Lipogenesis* | *FASN* | ACA AGC AGA ATT TGT CCA CCT TTA A | TCT CTA GAG GGC TTG CAC CAA |
|  | *SCD1* | TGC CCC TGC GGA TCT TC | TCG GGC CCA TTC GTA CAC |
| *Inflammation* | *CD11C/ Itgax* | CCA CTG TCT GCC TTC ATA TTC ATG | AAG ATG GCC CGG GTA CTC A |
|  | *CD68* | TGG TGT AGC CTA GCT GGT CTG A | GGC TAT AAG CGG TCC TAG CAA GA |
|  | *F4/80* | GGC CAT TGC CCA GAT TTT C | CGG TTG AGC AGA CAG TGA ATG A |
|  | *IL-1Β* | GCA ACT GTT CCT GAA CTC AAC T | ATC TTT TGG GGT CCG TCA ACT |
|  | *IL-6* | TCG GAG GCT TAA TTA CAC ATG TTC | TGC CAT TGC ACA ACT CTT TTC T |
|  | *MCP-1/ CCL2* | CTC TCT CTT CCT CCA CCA CCA T | CCA GCC GGC AAC TGT GA |
|  | *NOS2* | CAG CTG GGC TGT ACA AAC CTT | CAT TGG AAG TGA AGC GTT TCG |
|  | *TNF-Α* | TGG CCT CCC TCT CAT CAG TT | CAG GCT TGT CAC TCG AAT TTT G |
| *ER stress* | *ATF4* | GCT GAA AAA GAT GGA GCA AAA CA | TGC TCA GCC CGC TTC TTC T |
|  | *CHOP* | GCA TGA ACA GTG GGC ATC AC | CGA TGG TGC TGG GTA CAC TTC |
|  | *GRP78/ Bip* | ACC CTT ACT CGG GCC AAA TT | GCT TCA TGG TAG AGC GGA ACA |
|  | *spliced XBP1* | CTG AGT CCG CAG CAG GT | TGT CAG AGT CCA TGG GAA GA |
| *Endogenous control* | *18s rRNA* | AAC CCG TTG AAC CCC ATT | CCA TCC AAT CGG TAG TAG CG |

^1^ *ATF4*, Activating transcription factor 4; *CD11C/Itgax*, Integrin alpha X; *CD68*, CD68 antigen; *CEBPα*, CCAAT/enhancer binding protein alpha; *CEBPβ*, CCAAT/enhancer binding protein beta; *CHOP*, C/EBP homologous protein; *F4/80*, Adhesion G protein-coupled receptor E1; *FASN*, Fatty acid synthase; *GRP78/Bip*, 78-kDa glucose-regulated protein/binding immunoglobulin protein; *IL-1β*, Interleukin-1β; *IL-6*, Interleukin-6; *SCD1*, *MCP-1/CCL2*, monocyte chemotactic protein-1/ chemokine (C-C motif) ligand 2; *NOS2*, Nitric oxide synthase 2, inducible; Stearoyl-CoA desaturase-1; *TNF-α*, Tumor necrosis factor-α; *PPARγ*, Peroxisome proliferator-activated receptor γ; *spliced XBP1*, spliced X-box binding protein 1; *18s rRNA*, 18s ribosomal RNA.

**Table S3.** **List of antibodies used in western blotting analysis**

| **Protein** | **Company** | **Product number** |
| --- | --- | --- |
| CHOP | Santa Cruz Biotechnology | #2895 |
| eIF2α | Cell Signaling Technologies | #9722 |
| phosphorylated eIF2α | Cell Signaling Technologies | #3398 |
| HSC70 | Santa Cruz Biotechnology | #sc-7298 |
| IL-6 | Santa Cruz Biotechnology | #sc-32296 |
| JNK | Cell Signaling Technologies | #9252 |
| phosphorylated JNK | Cell Signaling Technologies | #4668 |
| NOS2 | Santa Cruz Biotechnology | #sc-7271 |
| TNF-α | Santa Cruz Biotechnology | #sc-52746 |
